# Long-Term and Seasonal Dynamics of Dengue in Iquitos, Peru

**DOI:** 10.1371/journal.pntd.0003003

**Published:** 2014-07-17

**Authors:** Steven T. Stoddard, Helen J. Wearing, Robert C. Reiner, Amy C. Morrison, Helvio Astete, Stalin Vilcarromero, Carlos Alvarez, Cesar Ramal-Asayag, Moises Sihuincha, Claudio Rocha, Eric S. Halsey, Thomas W. Scott, Tadeusz J. Kochel, Brett M. Forshey

**Affiliations:** 1 Department of Entomology and Nematology, University of California, Davis, Davis, California, United States of America; 2 Fogarty International Center, National Institutes of Health, Bethesda, Maryland, United States of America; 3 University of New Mexico, Albuquerque, Albuquerque, New Mexico, United States of America; 4 U.S. Naval Medical Research Unit No. 6, Lima, Peru; 5 Loreto Regional Health Department, Iquitos, Peru; 6 Hospital Regional Iquitos, Iquitos, Peru; 7 Hospital Apoyo Iquitos, Iquitos, Peru; 8 U.S. Naval Medical Research Center, Silver Spring, Maryland, United States of America; Centers for Disease Control and Prevention, Puerto Rico, United States of America

## Abstract

**Introduction:**

Long-term disease surveillance data provide a basis for studying drivers of pathogen transmission dynamics. Dengue is a mosquito-borne disease caused by four distinct, but related, viruses (DENV-1-4) that potentially affect over half the world's population. Dengue incidence varies seasonally and on longer time scales, presumably driven by the interaction of climate and host susceptibility. Precise understanding of dengue dynamics is constrained, however, by the relative paucity of laboratory-confirmed longitudinal data.

**Methods:**

We studied 10 years (2000–2010) of laboratory-confirmed, clinic-based surveillance data collected in Iquitos, Peru. We characterized inter and intra-annual patterns of dengue dynamics on a weekly time scale using wavelet analysis. We explored the relationships of case counts to climatic variables with cross-correlation maps on annual and trimester bases.

**Findings:**

Transmission was dominated by single serotypes, first DENV-3 (2001–2007) then DENV-4 (2008–2010). After 2003, incidence fluctuated inter-annually with outbreaks usually occurring between October and April. We detected a strong positive autocorrelation in case counts at a lag of ∼70 weeks, indicating a shift in the timing of peak incidence year-to-year. All climatic variables showed modest seasonality and correlated weakly with the number of reported dengue cases across a range of time lags. Cases were reduced after citywide insecticide fumigation if conducted early in the transmission season.

**Conclusions:**

Dengue case counts peaked seasonally despite limited intra-annual variation in climate conditions. Contrary to expectations for this mosquito-borne disease, no climatic variable considered exhibited a strong relationship with transmission. Vector control operations did, however, appear to have a significant impact on transmission some years. Our results indicate that a complicated interplay of factors underlie DENV transmission in contexts such as Iquitos.

## Author Summary

Description of long-term temporal patterns in disease occurrence improves our understanding of pathogen transmission dynamics and facilitates predicting new epidemics. Dengue, the most prevalent mosquito-borne, viral disease of humans, typically varies seasonally and on longer, inter-annual time scales. In most studies of these patterns, however, only a fraction of putative dengue cases are confirmed with laboratory diagnostics. Here we analyzed 10 years of fully confirmed dengue cases reported to a sentinel surveillance system in Iquitos, Peru. We describe the inter and intra-annual patterns of weekly case counts and relate these to climate and local vector control efforts. We show that dengue case counts vary seasonally in Iquitos despite very little variation in key climatic conditions, such as temperature and humidity. Overall, transmission correlated poorly with climate regardless of time lag. In seasons when vector control was conducted early, there was an apparent decline in cases later that season. We speculate that the relationships between climatic conditions and transmission of DENV in Iquitos are complex and non-linear, and that other factors, such as herd immunity, virus diversity, and vector control efforts, play key roles determining the timing and intensity of transmission.

## Introduction

Dengue is a mosquito-borne disease common throughout the tropics and sub-tropics [1], [2]. It is caused by infection with any of four antigenically-distinct, but related, dengue viruses (DENV-1, 2, 3, and 4) in a human-mosquito transmission cycle. The anthropophilic mosquito, *Aedes aegypti*, is the predominant vector [3], [4]. The long-term patterns of dengue incidence have been studied at numerous endemic sites, especially in Southeast Asia [5]–[12] and the Americas [8], [13]–[15]. Results highlight intra-annual (seasonal) and inter-annual (across multiple years) signatures in transmission intensity [8], [10], [16], [17], as well as occasional abrupt shifts in the age of people with clinically apparent illness [18]. Conclusions from these studies are mixed, although in aggregate they highlight that dengue occurs across a diverse array of conditions and that the key drivers of transmission similarly vary across those different contexts [8], [10], [16]. Continued, detailed documentation of these temporal dengue patterns in different, endemic populations is useful for improving our understanding of DENV transmission and testing the link of key variables like temperature to components of the virus transmission cycle [10], [19]–[21]. With this goal in mind, here we examined the temporal patterns of laboratory-confirmed dengue cases over a 10-year period encompassing the introductions of two novel serotypes into the Amazonian city of Iquitos, Peru.

Despite their informational value, long-term disease data sets often lack detail because of the costs associated with detection of potential cases and laboratory-based diagnosis [22]. Furthermore, the symptoms associated with dengue fever are non-specific and can lead to misdiagnosis [23], [24]. Nevertheless, many surveillance systems report suspected cases with confirmation of only a small fraction. While severe, hospitalized cases are less prone to misdiagnosis and are usually laboratory confirmed, they typically represent only a small proportion of the total number of people infected [25]. Moreover, severe disease outcomes are influenced by a variety of intrinsic factors (e.g., virus virulence, host exposure history) [26] and not necessarily external drivers, such as climate conditions.

Limitations of many long-term dengue datasets analyzed to date [9], [13], [15], [e.g. 27]–[30], in addition to variation in reporting methods, increase the difficulty and reduce confidence in defining universal properties of dengue transmission dynamics [8]. Johansson et al. [16] concluded that results of these analyses are sometimes biologically implausible and confusing, such as a negative effect of increasing temperatures on transmission [see references in 16]. Because transmission is seasonal, it will correlate with other seasonal patterns even though there is no mechanistic link. Thus, any statistical analysis should be rigorously scrutinized from a biological perspective and, preferably, cross-validated with additional data. A recent study analyzed seasonal dengue in Ecuador using linear mixed models incorporating entomological, epidemiological, and climate data [15]. The investigators found important influences of climate and entomological indices on monthly dengue case counts. Nevertheless, even using new and improved modeling approaches, in aiming to fit a particular statistical model to temporal disease data–which is often aggregated–to predict transmission patterns over time, the analysis potentially obscures other features of the time series that might generate hypotheses about underlying mechanisms.

Here, we examined the seasonal patterns of dengue over a 10-year period in relation to climatic factors and citywide vector control efforts. Our analysis focused on laboratory-confirmed dengue fever cases reported to a surveillance network based in multiple health-care facilities in Iquitos, Peru. During the period of study, two novel DENV serotypes invaded Iquitos, which was already endemic for DENV. In response to the invasions and subsequent epidemics, the local ministry of health conducted citywide house-to-house insecticide fumigation campaigns to kill adult mosquitoes and reduce virus transmission. Our analyses indicate that, although climatic variables correlate weakly with variation in transmission intensity, mosquito control efforts do appear to curtail epidemics when properly applied.

## Methods

### Study area

Iquitos is a city of ∼377,000 inhabitants that sits at the confluence of the Nanay, Itaya, and Amazon Rivers in the department of Loreto in northeast Peru. Iquitos has been thoroughly described in previous publications [23], [31]–[35]. In 2000, as part of a collaborative effort between the Peruvian Ministry of Health and the U.S. Naval Medical Research Unit No. 6, a surveillance network was established in public and military hospitals and clinics throughout Iquitos. For most years, 12 or 13 health centers participated, representing predominantly urban and peri-urban areas in and around Iquitos. A core of 3 hospitals and 6 clinics consistently provided samples throughout the study. A few health centers discontinued participation mid-study but were replaced by other health centers from the same geographic area. Additional details are described in Forshey et al. [23].

### Ethics statement

All data collection was conducted under study protocol NMRCD.2000.0006, approved by the Naval Medical Research Center Institutional Review Board (Bethesda, MD) in compliance with all U.S. Federal regulations governing the protection of human subjects. In addition, the study protocol was reviewed and approved by health authorities in Peru (Dirección General de Epidemiología). Written consent was obtained from participants 18 years of age and older. For participants younger than 18 years, written consent was obtained from a parent or legal guardian. Additionally, written assent was obtained from participants between 8 and 17 years of age. Prior to analysis, all data were de-identified and aggregated into weekly case counts.

### Study design

Details of the surveillance system, including inclusion criteria and laboratory assays are detailed in Forshey et al. [23]. Briefly, consenting participants (≥5 years old) provided an acute blood sample on the day they visited the health care facility for laboratory confirmation of DENV infection. Laboratory procedures included RT-PCR and virus isolation to identify acute infections and IgM ELISA to detect anti-DENV antibodies consistent with a recent infection. Convalescent samples collected 10 days to 4 weeks later were tested for anti-DENV IgM by ELISA. We identified the infecting serotype when possible (55%); positive diagnosis was generically defined a “DENV” infection when based solely on IgM assay results (Table 1).

**Table 1:** Summary of clinic case reports and citywide fumigations by transmission season.

		Reported casesΔ							Interventions	
Season	Years	DENV-1	DENV-2	DENV-3	DENV-4	DENV	All dengue	Negative	Dates	Effort (houses)
1	2000–2001	1	0	0	0	8	9	647		
2	2001–2002	59	9	122	0	234	424	770		
3	2002–2003	3	0	322	0	377	702	875	10/23/02 to 2/10/03	55,743
4	2003–2004	0	0	94	0	129	223	890		
5*	2004–2005	0	0	417	0	490	907	1,133	12/1/04 to 1/5/05	35,572
6	2005–2006	3	0	296	0	285	584	915		
7	2006–2007	0	0	185	0	100	285	721		
8	2007–2008	0	0	457	4	227	688	600	12/27/07 to 3/8/08†	33,363
9	2008–2009	0	0	21	504	300	825	528	10/20 to 11/3/08 and 2/5 to 2/21/09	24816; 32350
10	2009–2010	0	0	0	223	125	348	528	3/6/10 to 3/27/10	33,314
	**Sum**	**66**	**9**	**1,914**	**731**	**2275** a	**4995** b	**7,607**		

Δ , All reported cases were laboratory confirmed by PCR and/or IgM ELISA.

*, Case numbers adjusted for extended surveillance at one hospital in December 2004.

† , Two separate fumigation campaigns were conducted, separated by a week.

a , 45% of all DENV cases.

b , DENV cases were 40% of all reported cases (consenting participants).

### Vector control

In response to dengue outbreaks in Iquitos over the period of study, the Loreto Regional Health Department (LRHD) conducted large-scale vector control interventions (Table 1). In these, they sprayed inside houses with an ultra low volume (ULV), non-residual insecticide (deltamethrin [2002–2006], cypermethrin [2006–2008], or alpha-cypermethrin [2008–2010]) three times over approximately a three-week period. The LRHD attempts to treat all houses within designated sectors of the city, which are chosen based on epidemiological information (Table 1). These citywide efforts usually treated ∼40% of all houses in Iquitos, which total ∼80,000 houses. Data on interventions were provided by the LRHD. For our analyses we identified weeks when fumigation was conducted in the city and examined whether treatments were associated with reductions in dengue incidence within and across years.

### Analyses

Case data were restricted to the period between 1 July 2000 and 30 June 2010. Positive cases were those with evidence of virus (RT-PCR or virus isolation) or immunologic evidence of recent infection (acute or convalescent IgM ELISA titer>1∶100). We combined all DENV+ cases into weekly totals for use in correlation and wavelet analyses (see below).

Generally, people visiting health centers were received for 5–7 hours a day, 5 days a week, although there was some variability in rates across seasons and clinics. A major exception was a 2-week period of 2004 when surveillance in one hospital was extended to 24 hours a day due to the large number of dengue cases they were receiving. To correct for this extended effort, we rescaled the number of cases captured in these 2 weeks by the ratio of the maximum number of negative cases observed in the remainder of the time-series to the number of negative cases observed during those particular weeks (approximately 1∶5). For disaggregated analyses, the data were randomly thinned in these two weeks based on the same scaling factors.

Using the corrected time-series, we conducted autocorrelation analysis to characterize the temporal structure of the case data. Subsequently, we used wavelet analysis to identify temporal variation in the periodicity of dengue case reports. Our analysis was conducted on the square-root transformed and normalized (by standard deviation) time series using the Morlet wavelet transform and implemented in Matlab using the algorithm of Torrence and Compo [36].

Daily climate data for Iquitos was acquired from a US National Oceanic and Atmospheric Administration (NOAA) weather station located at the Iquitos airport. Reported variables include: mean, maximum, and minimum temperatures; precipitation; air pressure; wind speed; and dew point. From these data we generated several derived variables, including: daily temperature range (DTR; max - min), degree-days (DD), relative humidity (RH; 100 - 5*(Temp_mean - dewpoint)), and precipitation events (per week). We calculated degree-days using the triangle method and a 24°C threshold temperature for virus replication [DD 24]; [ 37,38]. We considered the river depth of the Amazon River as a covariate, because this variable changes dramatically over the course of the year as a function of rainfall in the Andes Mountains. At high river levels, fringe areas of Iquitos have occasionally flooded, which could have had an impact on mosquito populations. It is more probable, however, that river depth serves as an indicator of broader scale climate patterns that might correspond with conditions suitable for DENV transmission. River depth data for the Amazon River in meters above sea level was provided by the Servicio Nacional de Metereologia e Hidrologia, Peru.

Seasonal and annual climate patterns were summarized graphically using a loess smoother, which summarizes the data by fitting a local polynomial [39]. The degree of smoothing desired is controlled by the parameter α, where large values indicate more smoothing. We heuristically chose values of α to emphasize short-term and long-term temporal patterns in the data.

Because climate variables are highly collinear, interpretation of the relationship between any single variable and epidemiological patterns could be misleading. Maximum and minimum temperatures, for instance, should correlate in time. To address this issue, we conducted principal components analysis (PCA) on the climate variables. Briefly, PCA reorients a set of n covariates into n principal components (PCs) based on their covariation structure. The first PC (PC1) always captures the largest proportion of the covariance between the covariates, with successive PCs explaining less and less of the remaining variation. With more correlation among covariates, fewer PCs are required to capture most of the variation in the dataset. By definition, the resulting principal components are orthogonal with each other (i.e., they do not correlate) and the set of n PCs exactly encapsulates all covariation among the covariates. Within a PC, loading values describe the relative contribution of each original covariate. Higher loadings indicate greater correlation and high loadings on the first few PCs indicates the overall importance of that covariate in the covariation structure of the dataset.

We examined the relationships between weekly DENV cases and climate variables using cross-correlation maps [CCMs; 40]. For each variable, maps were generated by varying the temporal lag and the period over which the variable was aggregated. Briefly, cases in week t_0_ were correlated with each covariate aggregated over a range of weeks prior to t_0_, defined by the interval [t_0_-a, t_0_-b]. We evaluated the mean, median, and maximum values of the covariate, and, in some cases, the sum for each period. We present results for the median unless the sum was more appropriate. For example, it is possible that rain influences dengue cases 4–8 weeks later because of effects on mosquito population dynamics. In that case, we set a = 4, b = 8 and looked at the correlation of maximum rainfall over that interval with the number of dengue cases a month in the future. To limit the identification of spurious correlations, we did not investigate lags more than half a year before the cases were observed (27 weeks). We believe, however, that effects most likely to have biological relevance on transmission would occur within a lag of 17 weeks (1 trimester). To investigate both linear and monotonic associations between climatic variables and cases we calculated Pearson and Spearman correlations. We categorized the correlation coefficient, *r*, as follows: *|r|*<0.1, no correlation; 0.1≤|*r*|<0.2, very weak, 0.2≤|*r*|>0.3, weak; 0.3≤|*r*|<0.4, weak moderate; 0.4≤|*r*|<0.5, moderate; |*r*|≥0.5, moderate strong to strong. Because of the large number of tests conducted (each CCM equates to 338 correlation tests), we did not calculate p-values and rather focused on the relative strength of correlations. Unless otherwise stated, all analyses were conducted with R 2.13.

## Results

### Dengue patterns

**Figure 1:**
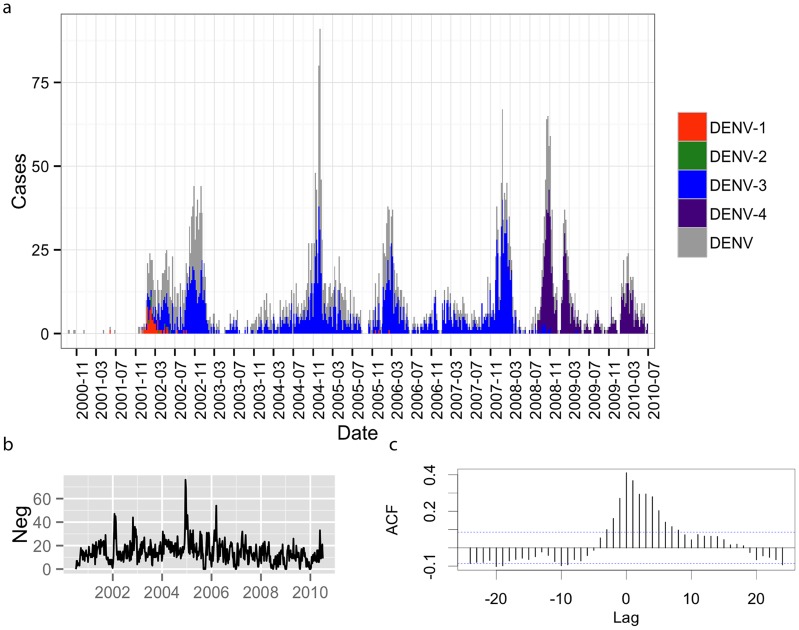
Cases captured by clinic-based surveillance system in Iquitos, Peru between 2000–2010. *a*, Dengue cases by week, serotype indicated where possible. *b*, non-dengue cases. Note elevated effort in December of 2004. *c*, Cross-correlation plot between dengue cases and non-dengue cases showing that these were correlated. The strongest correlation was at a lag of 0.

**Figure 2:**
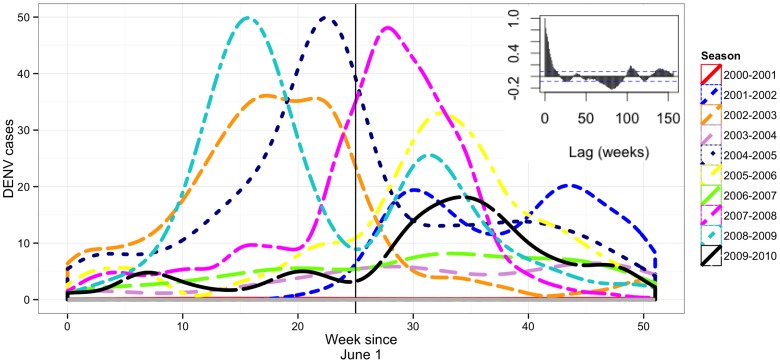
Dengue epidemics overlaid to illustrate shifting of peak incidence. The epidemics are centered on the last week of December (black vertical line). Inset: autocorrelation of cases across the entire time-series showing significant negative autocorrelation around a lag of 70 weeks.

Over the 10 years of study, 12,602 febrile participants were enrolled, 40% of whom were laboratory diagnosed as having acute or recent DENV infection (Table 1). Although very few dengue cases were detected at the beginning of the study (consistent with serology data [34]), after late 2001, outbreaks occurred on an annual basis (Figs. 1, 2). Overall, weekly case reports fluctuated seasonally (i.e., DENV positive and negative cases together; Fig. 1). Cross-correlation analysis showed that the number of cases diagnosed as something other than dengue (DENV negative cases) mirrored the number of dengue positive reports (i.e., the best lag was 0; Fig. 1c).

Wavelet analysis indicated that the annual periodicity in transmission was particularly strong from the 2004–2005 season forward (Fig. S1). A longer, ∼3 year periodicity was also suggested by the analysis, but the 10 year time-series was too short to place confidence in this result. Over all years, 75% of DENV cases were reported between the 37^th^ week of the preceding year and 13^th^ week of the subsequent year, peaking on average in the last week of December (Fig. 2). We thus define the dengue season in Iquitos as occurring between September and April (between trimester III and trimester I of the subsequent year).

Over the 10 dengue seasons single serotypes accounted for the majority of all cases. DENV-1 was dominant in the first season, followed by the emergence of DENV-3 in 2001, [34], [genotype III;41], and the emergence of DENV-4 in 2008 [genotype II; 42](Fig. 1, Table 1). DENV-2 (lineage I of American/Asian genotype) was only detected in a few study participants in 2001–2002. DENV-1 appeared at low levels in 2002–03 and 2005–06 when DENV-3 was dominant.

Although transmission intensified on an annual basis, the magnitude and timing of the peaks varied across seasons. Temporal autocorrelation of the number of weekly DENV cases indicates a strong positive auto-correlation at a lag around 2 years and a negative correlation around a lag of 1.5 years (Fig. 2). This result is consistent with an apparent shift in the timing of peak transmission from year to year (Fig. 2). In other words, the inter-epidemic period fluctuated between approximately 8 and 16 months.

### Climatic patterns

Climatic variables demonstrated seasonality in Iquitos, although the magnitude of variation was small (Table 2, see SI). Maximum and mean weekly temperatures were warmest in trimesters III and I (between November and April), coinciding with the timing of detection of most dengue cases (Figs. S2, S4). Mean and minimum temperatures showed a gradual increasing trend over the 10 years, culminating in a ∼1°C increase between 2000 and 2010 (Fig. S4, S6). Cumulative weekly DD_24_ largely mirrored trends in mean and maximum temperatures, peaking in late trimester III (November [28.04°C•days]; Fig. S8) and bottoming in trimester II (June [19.27 C°•days]). The 10-year trend in DD_24_ was highly non-linear, lowest in early 2008 and increasing rapidly to its highest levels in 2009 and 2010 (Fig. S8). Precipitation occurred throughout the year, but it was usually lower in the later part of trimester II (July [3.77 cm•week^−1^] and August [4.66 cm•week^−1^]; See Table 2, Fig. S12). Over all years, rainfall amounts were highest between 2003 and 2008, dropping significantly in later years, although the number of precipitation events remained the same (Fig. S14). Additional climatic variables are shown in the SI.

**Table 2:** Summary of climatic variables by trimester (annual). Values are the mean ± 1 SD.

	I	II	III
**Maximum daily temperature (°C)**	32.31±1.09	31.65±1.22	33.06±0.98
**Mean daily temperature (°C)**	26.10±0.66	25.37±0.77	26.22±0.65
**Minimum daily temperature (°C)**	22.58±0.57	21.57±0.91	22.32±0.69
**DD_24_ (°C)**	25.09±4.20	20.83±4.37	26.97±3.67
**DTR (°C)**	9.74±1.15	10.07±1.29	10.75±1.21
**Precipitation (cm)**	7.57±6.47	5.65±6.37	7.06±6.21
**Precipitation events**	3.85±1.43	3.45±1.45	3.39±1.29
**Relative humidity (%)**	84.59±3.15	85.10±2.82	83.19±2.83
**Wind speed**	2.35±0.62	2.03±0.57	2.45±0.52
**Amazon river level (m.a.s.l.)**	114.95±1.38	113.62±2.59	110.85±2.08

Because climate variables correlate, we conducted principal components analysis (PCA) to simplify the data and identify subsets of highly collinear drivers. The results of the PCA identified three components that described 79% of the variation among the climate variables (Table 3). The first, PC1, related most strongly to temperature variables and humidity. PC1 increased with increasing humidity and decreased with increasing temperatures. The second, PC2, captured variability in temperatures. PC2 decreased with increasing minimum temperature and river level and increased with larger DTR. The third component, PC3, decreased with precipitation and wind speed and increased with river level (Table 3). All three principal components exhibited seasonal periodicity, although this was attenuated for PC3 in later years. (Fig. S21, S23, S25).

**Table 3:** Variable loadings from principal components analysis.

Variable	Components		
	PC1	PC2	PC3
**Max temperature**	−0.48	0.0476	0.0930
**DD_24_**	−0.466	−0.176	0.0849
**Mean temperature**	−0.431	−0.239	0.229
**DTR**	−0.354	0.450	0.0268
**Min temperature**	−0.171	−0.639	0.0999
**Wind**	−0.150	−0.228	−0.671
**Precipitation (cm)**	0.102	−0.272	−0.522
**River level (m.a.s.l.)**	0.195	−0.402	0.381
**Relative humidity**	0.378	−0.11	0.232

Taken together, conditions in Iquitos can be described by three seasons: In trimester I, temperatures are warm, rainfall is elevated, the level of the Amazon river is increasing and dengue cases subside; in trimester II, conditions are relatively cooler and drier, the river begins to subside, and there are few dengue cases; in trimester III temperatures are their warmest and precipitation increases, the river subsides to its lowest levels, begins to rise again, and dengue transmission picks up.

### Dengue and climate

**Figure 3:**
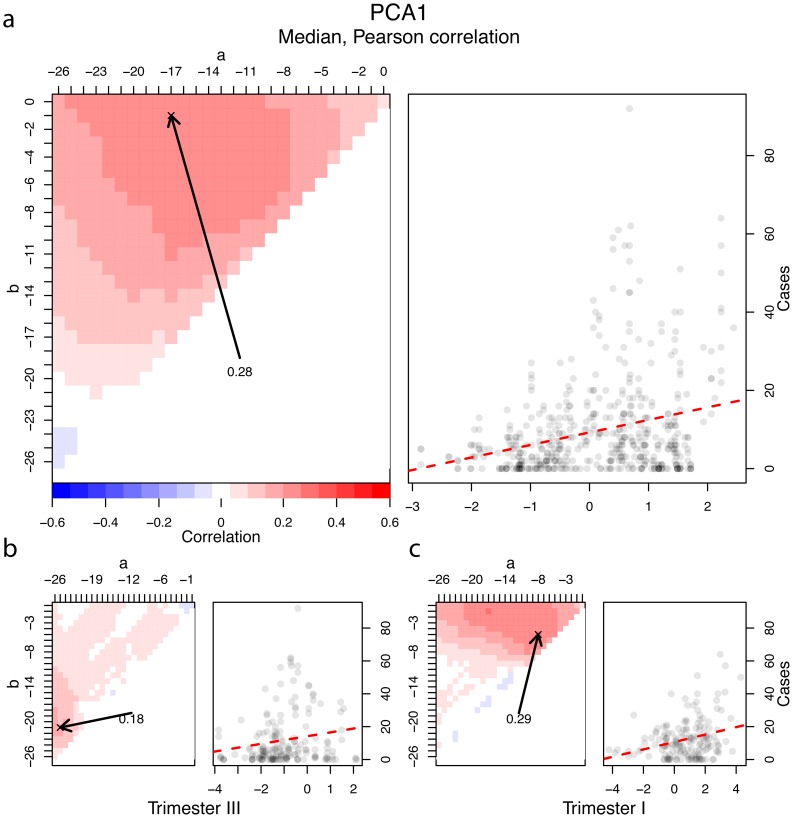
Correlating weekly dengue cases to climatic variables. *A*, left panel: A cross-correlation map relating weekly dengue cases to PC1 on an annual basis (Pearson correlation). The median value of weekly PC1 scores most strongly correlated with dengue cases over the previous 17 weeks (a = −17, b = −1; See Methods, Analyses). *A*, right panel: Scatterplot of weekly cases and median PC1 at the corresponding lag. Points are transparent to illustrate point density. Dashed line illustrates the linear trend in the data, although the relationship does not appear to be strictly linear; most weeks with high case counts occurred when PC1>0. *B*, trimester III. *C*, trimester II. Layout in *B* and *C* same as *A*. CCMs and scatterplots for all other climate covariates and principal components are in the SI and summarized in Fig. 4.

**Figure 4:**
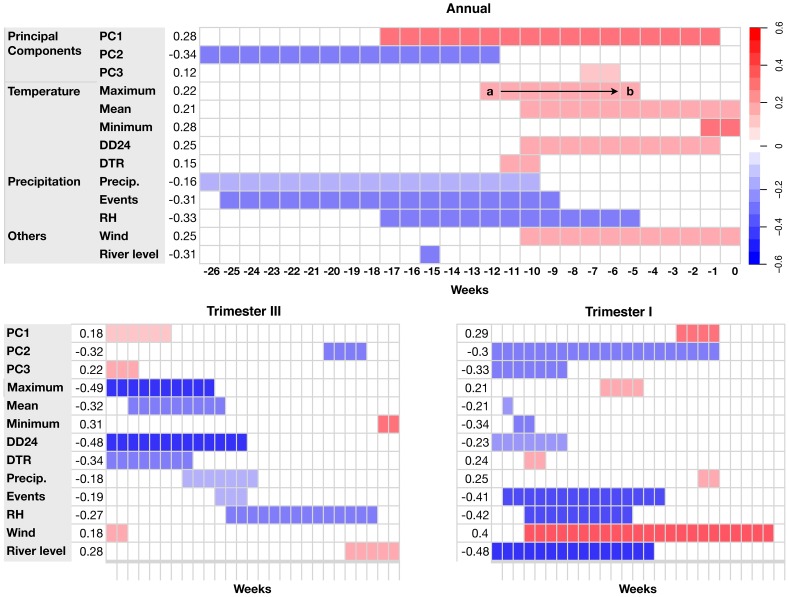
Summary of CCM results for each climate covariate and principal components on an annual basis and for trimesters III and I. The period (denoted by the arrow between the a and b in the figure) over which a covariate most strongly correlated with current dengue cases is colored according to the strength of the correlation (*r_Pearson_*; legend same as Fig. 3), with blue indicating negative correlation and red indicating positive correlation. See Figure S28 for Spearman correlation results.

We related weekly reported dengue cases to climate variables using temporal cross-correlation maps (CCMs; Fig. 3; see Methods and SI). Because pair-wise relationships to individual climate variables can be misleading and conflated by collinearity between climate variables, we first examined CCMs of the three principal components described earlier. We subsequently considered specific individual variables commonly associated with DENV transmission. In all instances, we produced CCMs for the whole year and for trimesters I and III (Fig. 3), when most DENV transmission took place (see above). Overall, CCMs showed that there was a correlation between most climatic variables or their components and reported dengue cases, although the correlations—especially on an annual basis—were often weak (|*r*|<0.3; Fig. 4). For each CCM, we identified the maximum absolute *r* and plotted weekly case reports against the climate covariate to characterize the nature of the relationship (linear, non-linear; Fig. 3).

We first examined the relationship between weekly dengue reports and the first three principal components, which consolidate highly collinear variables into orthogonal components (Table 3). The first component, PC1, which associated negatively with temperature variables and positively with RH, correlated weakly and positively with dengue cases when aggregated over a broad period from 17 to 1 week earlier (Figs. 3, 4, S23). This means that a period of relatively lower temperatures and elevated RH preceded high case counts. When we focused only on trimester III, the correlation was weaker (0.18) and the lag was greater ([−26, −21]; Figs. 3, 4). In trimester I, the correlation was stronger (0.29) and the lag was less ([−9, −6]; Figs. 3, 4). The second component, PC2, aggregated over 26 to 12 weeks prior, correlated more strongly (weak moderate) with cases on an annual basis (−0.34, [−26, −12]; Figs. 4, S25). In the principal components analysis, PC2 correlated most strongly with minimum temperatures and DTR, thus when minimum temperature was high and DTR was small 3–6 months previous, case counts were elevated (Table 3). In trimester III, PC2 again correlated negatively (−0.32) with cases, but at a smaller lag ([−6, −2]; Figs. 4, S25). The PC2 correlation and lag for trimester I was similar to the annual pattern (−0.3, [−26, −6]; Figs. 4, S25). Finally, PC3, which correlated most strongly with wind speed and river level, showed a weak correlation with cases on an annual basis (0.12, [−7, −6]; Figs. 4, S27). In trimester III, PC3 correlated weakly and positively at a large lag (0.22, [−26, −24]). In trimester I, the correlation was negative and strongest at a large lag as well (−0.33, [−26, −20]; Figs. 4, S27). PC3 also correlated positively with cases at shorter, biologically relevant lags in this trimester (Fig. S27). Pearson and Spearman correlations for PC1 and PC2 were similar (Fig. S28). PC3, however, differed markedly in annual and trimester III CCMs (Figs. S27, S28).

Within what we considered a biologically relevant window of 17 weeks, PCs 1 and 2 correlated with cases on an annual basis. By trimester, only PC2 correlated significantly in trimester III and both PC1 and PC2 correlated in trimester I, although the relationship with PC2 was distributed over a broad range of lags.

Examination of scatterplots relating components to weekly cases revealed distinct non-linear patterns. The number of cases increased more rapidly with increasing PC1 [−17, −1] than expected of a linear relationship (Fig. 3). There was a considerable increase in variation in the number of cases each week at higher values of PC1 (i.e., at lower maximum/mean temperatures and increasing humidity). Thus, few cases should be expected when PC1 is low 1 to 17 weeks earlier, but it is uncertain how many cases will result when PC1 is elevated over the same period. The patterns by trimester were mostly similar. Conversely, the number of cases decreased more rapidly than expected (linear) in relation to increasing PC2 (Fig. S25). The scatterplot of cases against the best PC2 lag shows a decrease in both the mean and variance of cases as PC2 increases, indicating that the weeks of highest incidence occurred when PC2 was very low (high minimum temperature, low DTR; Table 3) between 26 and 15 weeks before. As with the relationship between PC1 and cases, due to heteroskedasticity, high values of PC2 always correspond to few cases. The patterns were similar by trimester, except the lag was much less in trimester III. Finally, the scatter plot of cases relative to PC3 showed a distinct humped pattern with most transmission occurring when PC3 was between −0.5 and 0.5, suggesting that there is a stronger association between this component and cases than that measured with simple correlation (Fig. S27). Partitioning this analysis by trimester partly resolved this non-linearity: in trimester III the relationship is positive and linear while in trimester I it is negative and linear (Fig. S27).

Mosquito development and virus replication in the mosquito are temperature dependent [43], so ambient temperatures are often thought to play an important role in DENV transmission [37], [44]. Precipitation, too, is often thought to be a key local variable influencing DENV transmission because mosquitoes require aquatic habitats for larval development [8], [43]. Relative humidity combines aspects of temperature and precipitation and is probably directly important for mosquito survival because it influences desiccation rates. All of these variables naturally correlate with each other and for this reason we focused on the analysis of principal components. When considering individual variables, however, we found that correlations on an annual basis were mostly weak (Fig. 4; See the SI for results, figures S8—S27). The number of precipitation events and relative humidity correlated strongest at relatively large lags (Fig. 4). Several individual variables, temperature related variables in particular, correlated with case reports within a 17-week lag (Fig. 4). In trimester III, maximum temperature and DD_24_ showed moderate negative correlations, but at very large lags. Within our biologically relevant window of 17 weeks, only minimum temperature, RH and river level showed appreciable correlations in this trimester (Fig. 4). In trimester I, precipitation events, RH, wind, and river level were most strongly correlated with weekly case numbers, but at large lags. Only precipitation and wind speed correlated within a lag of 17 weeks (Fig. 4).

There was evidence of non-linear relationships and heteroskedasticity in many instances (see, for example, mean temperature in Fig. S5). These were occasionally resolved when portioning the analysis by trimester. That is, a positive relationship in trimester III changed to a negative relationship in trimester I.

On an annual basis, results for Spearman correlations were largely similar to those for Pearson correlations, although the correlations were stronger and extended over a longer period for temperature covariates (Fig. S28). The one exception was DTR, which correlated positively in Pearson tests, but negatively in Spearman tests at a shorter lag—although in both cases the correlation was very weak and may not be important (Fig. S11). On a trimester basis, several variables correlated well with weekly DENV cases within a 17-week lag. These were, for trimester III, minimum temperature (0.39 [−2, 0]), DTR (−0.44 [−8, 0]), and RH (−0.42 [−15, −4]; Fig. S28).

### Vector control

**Figure 5:**
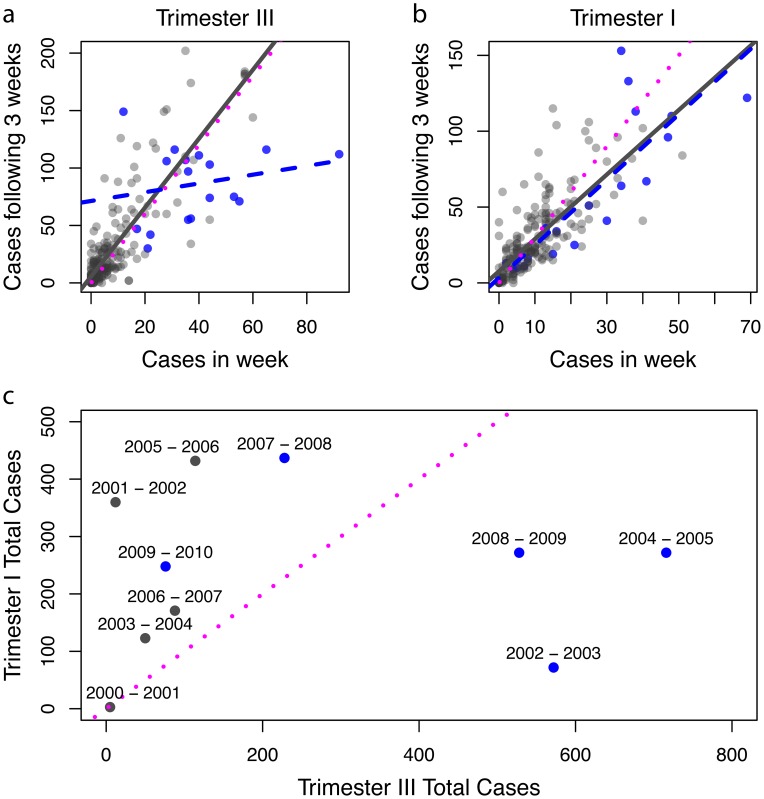
Control effects. *A–B*, The number of dengue cases in a week are plotted against the total number of cases in the subsequent three weeks. The red line corresponds to the following three weeks all having the same number of cases as the initial week (i.e. the slope is 3); the black line is the trend for weeks with no fumigation; the blue dashed line and blue points are for weeks when fumigations were conducted. Note that in *B* the black line has a smaller slope than the red line, indicating a natural decline of case numbers week-to-week. *C*, control and seasons summarized. The red line is the 1∶1 line indicating an equal number of cases in the first and second half of the dengue season, blue dots are seasons with interventions; black dots are seasons when no intervention was conducted. See Table 1.

In addition to climatic variation, city-wide efforts to fumigate households with insecticide to curtail transmission hold large potential for shaping inter and intra-annual patterns of transmission in Iquitos. Using data provided by the LRHD on their vector control efforts, we assessed the potential effect of citywide interventions on the number of reported dengue cases by plotting cases in week t_0_ with the total number of cases in the subsequent 3 weeks. We split the data by whether an intervention was taking place in week t_0_ and by trimester (Fig. 5). As indicated above, in trimester III dengue outbreaks were usually beginning and so the relation between cases this week and cases over the following three weeks was approximately 1∶1 or greater (compare black and red lines in Fig. 5). In seasons when an intervention was conducted in trimester III (blue points), however, the relation was less than 1∶1, which indicates a reduction in the rate new cases were captured. Conversely, in trimester I transmission was subsiding and the relationship was usually less than 1∶1 even in the absence of vector interventions. Moreover, there did not appear to be any impact of interventions when they were conducted in trimester I (compare black and blue lines). That is, when interventions were conducted in trimester I any reduction in transmission was masked by the natural decline in the number of new cases reported. Over the full 10 year study period, when transmission and interventions both occurred in trimester III there appeared to be lower transmission in the subsequent trimester I (Fig. 5). We did not observe any seasons with high trimester III transmission without any intervention activities.

## Discussion

### Summary of findings

Dengue was not reported in Iquitos from the late 1970s—the end of the hemisphere-wide campaign to eradicate *Ae. aegypti* from the Americas—until a DENV-1 outbreak in 1990 [45]. Continuous DENV transmission has been detected since that time. DENV-2 American was detected in 1995 [46]. Over the period of this study, 2000–2010, DENV-3 [34] and then DENV-4 [42] invaded the city. DENV-3 was dominant over 6 transmission seasons until it was replaced by DENV-4 in 2008 [17]. Virus transmission dynamics in Iquitos have, therefore, been largely due to single serotypes and marked by annual periodicity, suggestive of seasonal forcing. The magnitude and timing of outbreaks were variable from year to year. Because of the obvious seasonality of dengue in Iquitos and elsewhere, we examined the role of climatic drivers in transmission dynamics. Our descriptive analysis of temporal variation in dengue cases in relation to climate did not, however, resolve clear relationships. The magnitude of seasonal climatic variation in Iquitos was quite small and at least low-level transmission was detected year-round. On an annual basis, almost all of the climatic variables we considered correlated weakly (|*r*|<0.3) with the number of dengue cases reported each week, with a few exceptions that were only slightly better correlated (e.g., relative humidity). Partitioning the analysis by trimester revealed stronger relationships, but most of these were distributed over very long lags (>20 weeks), suggesting that the observed correlation was due to the phase difference between seasonal signals and not a mechanistic link. Principal components analysis facilitated interpretation of the observed patterns, but generally highlighted that the relationship between climate and dengue in a place like Iquitos—where climate conditions may be suitable for transmission year-round—is complex, with no single dominant climate driver. Finally, citywide vector control efforts targeting adult mosquitoes—depending on their timing—appeared to reduce transmission.

### Implications and context

In many regions of the world, particularly Southeast Asia, dengue epidemiology is characterized by co-circulation of multiple serotypes [47]. Serotype co-circulation complicates analysis of disease dynamics because the different virus serotypes interact immunologically at the level of the host and may be differentially transmitted by local mosquito vectors [25], [48]–[50]. After examining laboratory-confirmed dengue cases reporting to a network of clinics and hospitals in Iquitos, Peru, we provide a different perspective on the dynamics of this disease from that reported for other contexts. In this isolated population of ∼400,000, transmission has largely been dominated by single serotypes. On a few occasions, a small fraction of cases were due to other serotypes. Because of its population size [13], circulation of a single serotype (and genotype of a serotype) over multiple years and at least one confirmed dengue case in the majority of weeks (81%), we conclude that DENV is endemic and persists in Iquitos year-round. Dengue is not hyper-endemic (i.e., stable, year-to-year, co-circulation of multiple serotypes [47]), probably because of limited connectivity to other dengue endemic areas. Occasionally, new virus strains are amplified in other parts of Peru, Colombia and/or Brazil, from which they are introduced and become established in Iquitos. Indeed, the molecular epidemiology and timing of DENV-3 and DENV-4 emergence [41], [42] suggests that those viruses arrived to Iquitos via the Peruvian cities of Pucallpa to the south (population ∼120,000; DENV-3) and Yurimaguas to the southwest (population ∼48,000; DENV-4) both separated from Iquitos by a short flight or multi-day boat ride. There are no roads connecting those cities to Iquitos.

Our data show that dengue incidence in Iquitos follows a clear, seasonal pattern with the number of dengue cases peaking around December (calendar year trimesters III and I in this analysis). The timing of this peak varied year to year such that a short inter-epidemic period appeared to be followed by long inter-epidemic period. While this pattern is intriguing, our time series was too short to determine whether it is real and not a coincidence. Wavelet analysis suggests a 3-year cycle in incidence similar to that reported for hyper-endemic settings [27], but, again, 10 years is insufficient data to confirm this result statistically.

We find it compelling that transmission was distinctly seasonal, especially after 2004, even though the magnitude of seasonal variation in climate was very small. When looked at on an annual basis, PC2, which aggregated minimum temperature, DTR, and river level, was the best linear covariate. This correlation, however, was distributed over large lags and so may simply be the result of the phase difference between two seasonal signals. PC1, which aggregated temperature variables and RH, showed some correlation with cases and over shorter lags, but it was weak. PC3 showed a very weak linear correlation, but scatterplots indicated that the actual relationship was highly non-linear. PC3 aggregated precipitation and wind speed. When we partitioned the analysis by trimester, we observed that PC2 correlated with cases in a biologically reasonable time frame in trimester III and PC1 did so in trimester I. Neither of these correlations was very strong. Also, the non-linearity in the relationship between PC3 and weekly cases was partly resolved, i.e., the correlation was positive in trimester III and negative in trimester I. Altogether, the analysis of principle components with CCMs suggests, at best, weak climatic forcing of dengue transmission in Iquitos. This is confounded by the impacts of vector control (see below), herd immunity [17], [34], [51], and non-linearities in the relationships—in addition to the caveats associated with our analysis (see below).

Both RH and minimum temperatures have been cited elsewhere as strong correlates of DENV transmission [10], [13], [28], [29]. Precipitation, too, is commonly observed to drive transmission [15]. On an annual basis, temperature-related variables predominantly correlated with dengue cases within a 17-week lag. On a trimester basis, minimum temperature, RH, and river level stand out in trimester III (Fig. 4). Precipitation and wind speed stand out in trimester I. Spearman correlations highlighted minimum temperature and RH, but also DTR. Elevated minimum temperature could accelerate larval development and reduce the DENV extrinsic incubation period. Although RH has been shown to correlate positively with transmission [10], within the range of values we observed, it correlated negatively with cases. This is likely due to the relationship between RH and temperature (see Table 3). River level, which is driven by precipitation in the Andes mountains and not in Iquitos, probably serves as a proxy for some other proximate factors influencing local mosquito populations or transmission because it has limited impact in the areas of the city where dengue is most common. When river levels are high, transport times are significantly reduced (AC Morrison, personal communication) and *Ae. aegypti* abundances on boats are highest in October (Guagliardo et al. in review). Similarly, although wind speed might affect mosquito behavior, it seems more probably that wind proxies for other environmental conditions.

The range of temperatures experienced each day (DTR) may modify *Ae. aegypti* life history traits and *Ae. aegypti*-DENV interactions [44], [52]–[54]. In Thailand, large daily fluctuations corresponded with less transmission. On an annual basis, we found DTR to be weakly correlated with dengue cases. DTR did load heavily on PC2, which was more strongly correlated with transmission. This latter relationship indicates that high DTR over a period 3–6 months earlier correlated with high current case counts (Fig. 4). In trimester III, however, DTR was one of the strongest correlates over short lags in Spearman tests (Figs. S11, S28). While this result was not apparent in Pearson tests, it suggests that DTR may be epidemiologically important for DENV transmission in Iquitos, as suspected for parts of Thailand.

Overall, it appears that climatic conditions in Iquitos always hover near to a critical threshold for transmission. For instance, a small difference in temperatures could allow female mosquitoes to become infectious after only 2 gonotrophic cycles, as opposed to 3 or more, which would be expected to markedly increase vectorial capacity [55]. Clearly, though, other undefined factors are playing important roles in determining the temporal patterns of DENV transmission in Iquitos [17].

Although mosquito abundances must be important, we do not think that dengue seasonality (especially the increase in transmission) is uniquely driven by fluctuations in *Ae. aegypti* populations. *Aedes aegypti* is found in Iquitos year-round and, although population size fluctuates, is relatively abundant when dengue transmission is low [Reiner et al. unpublished 32]. This may contrast with other contexts where climatic variables, especially precipitation, vary more than in Iquitos [15]. On the other hand, our results indicate that vector control efforts targeting adult mosquitoes in large portions of the city were effective, accelerating virus fade-out when the intervention was applied early in the dengue season. In addition to truncating lifespan and killing infected and incubating mosquitoes, these control efforts may transiently reduce the vector population below a threshold density necessary for sustaining epidemic transmission.

Health authorities in Iquitos have responded to a number of outbreaks since 2000 with the intent to kill infected and/or infectious adult *Ae. aegypti* and reduce mosquito abundance (Table 1). Their interventions usually involved three cycles of non-residual, intra-domicile ULV space spraying with an adulticide (deltamethrin, cypermethrin, or alpha-cypermethrin). Spraying was organized by spatial units defined by the ministry of health and was typically guided by epidemiological information in order to prioritize areas with the largest number of cases. A large number of domiciles were usually treated over a period of several weeks to months. Our analyses indicate that these responses were effective at reducing transmission, which is most easily detected when cases peaked in trimester III and an intervention was conducted in this same period, i.e., early in the transmission season (Fig. 5). Later, in trimester I, it was more difficult to detect a reduction in the number of cases caused by fumigation efforts, presumably because transmission intensity was fading for reasons other than vector control. We assume conditions become less suitable for transmission, but cannot say whether this is due to the effects of temperature on virus replication, a natural reduction in the vector population (although vector abundances remain high in trimester I; [Reiner, et al. Unpublished]), increasing herd immunity or some combination of these and other factors.

We deliberately focused on characterizing the temporal patterns of dengue case reporting in Iquitos in relation to commonly studied covariates, namely climate variables. One of our major goals was to inform the development of mechanistic models. In doing so, we made two methodological observations. First, CCMs are a useful tool for describing the nature of a linear correlation between two covariates. In our case we used them to find the ‘best’ periods of correlation, but found also that the maps were often very “flat.” This simply indicates that the correlation was similar across a range of lags and periods. In other instances, there were clearly multiple possible solutions; i.e., there were several different ‘best’ lags. Second, when plotting the scatter plot of cases against covariates at the best lag and period, we found many non-linear patterns. PC3, which had no linear correlation with dengue case counts at any lag on an annual basis, exhibited a distinct humped relationship. Together, these observations bring into question interpretation and use of classical, linear modeling methods for fitting case data without first doing careful exploratory data analysis. Mixed modeling approaches incorporating appropriate lags and confounds might then prove appropriate tools for modeling and predicting transmission [15], [56]. Nevertheless, where the shape of relationships is uncertain, a priori, non-parametric methods such as general additive models would be more useful. New tools are needed for exploratory analysis, however, to search across lags in order to identify the periods when covariates are most strongly associated with the variable of interest, which will guide model development. Regardless, it is critical that we develop an improved understanding of the relationship between virus transmission dynamics [e.g. 17], [57], [58], per se, and disease.

### Caveats

Although the surveillance program that generated our data was largely uniform across the years of study, changes in personnel, protocol modifications, and variation in transmission intensity likely affected the number of cases captured on a daily basis by the system. Moreover, our surveillance only covered approximately 40% of the Iquitos population, participation rates were far short of 100%, and participation was only sought during the day. We specifically addressed one period of a large increase in surveillance effort during a particularly intense dengue outbreak, but otherwise did not attempt to correct for variation in case capture efficiency. We acknowledge this limitation and in our correlation analyses used a non-parametric method (Spearman correlations) that–for the most part–confirmed results from the Pearson correlations. Reporting rates probably varied over the 10 years as a function of disease severity and other factors influencing individual care-seeking behavior. Although each year there was an increase in the number of dengue cases each year, awareness both in the medical community and the general public would be expected to lag actual transmission. We speculate that care-seeking behavior may change during the course of a dengue outbreak. Initially, during the increase in DENV transmission, people may be more likely to report to a clinic or hospital at the first signs of a fever or other symptom. After a period of transmission and the recognition that dengue cannot be cured with a drug, people may self medicate mild disease with an antipyretic and, thus, be less likely to visit a clinic or hospital. We acknowledge that although all of the cases in our data set were laboratory confirmed, factors not associated with transmission per se likely influenced the patterns we observed and so we considered these patterns only indicators of the true transmission dynamic.

### Conclusions

In contrast to the seasonal patterns described above (i.e., transmission typically peaking in late December), we note that the DENV-1 outbreak in 1990 peaked in May [45] and the DENV-2 outbreak in 1995 peaked in August [46]. While climatic averages may have changed some since then, the seasonality has not, which begs explanation. In light of the weak, direct relationships between climatic variables and dengue case totals we measured and the observation that conditions in Iquitos may always support some level of transmission [10], we posit that other factors that we did not measure are important for determining the timing of intra-annual fluctuations and seasonal peaks in transmission. Both the 1990 and 1995 outbreaks were associated with novel virus introductions and we found no record of attempts during those times to perform citywide fumigation campaigns such as those begun in 2003. Because *Ae. aegypti*, is present year-round in Iquitos, herd immunity and the timing of virus introduction emerge as key determinants of when outbreaks occur. Consistent with this idea, DENV-3 transmission remained high in April/May of 2002, later than all other seasons in our analysis (Fig. 2). DENV-4, on the other hand, peaked in October of 2008. After invasion, as herd immunity rises, variation in mosquito abundances and the suitability of environmental conditions for transmission, should play more of a role determining transmission dynamics. We speculate that the timing and intensity of mosquito interventions to control mosquito populations influenced dynamics in subsequent seasons through their effect on herd immunity [59]. Our future work will focus on testing these hypotheses using mechanistic models [e.g.21].

We emphasize that while climate plays a key role in DENV transmission at broad spatial scales [8], [10], there remain significant uncertainties regarding its specific role and importance when weighed against other drivers at local, fine scales. In different geographic contexts, climate could play a greater role in DENV transmission than in Iquitos, highlighting that DENV ecology is complex and context dependent. Nevertheless, the patterns we document here provide valuable material for the development of mechanistic models that can be used to explore alternative hypotheses about transmission drivers in addition to climate. Importantly, our results indicate that vector control efforts, albeit intensive, can reduce transmission if timed and placed properly. This indicates that vector control can be an effective tool for preventing dengue.

## Supporting Information

Figure S1 The local wavelet power spectrum (sqrt-transformed, dark red = high, dark blue = low) corresponds to the amount of variation in the signal that is explained by different periods (y-axis) as a function of time (x-axis). The global wavelet spectrum averages this variation across time. Throughout most of the time series there is a strong annual component with some sub-annual components driven by differences in epidemic timing and a double peak during the 2008–09 season. Multi-annual components are present, on the order of 2–3 years, but the length of the time series is too short to provide strong support. Accounting for edge effects, the periods that can be detected at a particular time lie above the thick black line (cone of influence). The areas of statistically significant power at the 5% level are contoured by the thin black lines. On the global wavelet spectrum, this threshold is indicated by the dashed line. (a) result assuming uncorrelated, white noise; (b) result assuming autocorrelated, red noise. Note in (b) the two novel serotype introductions are emphasized (2001–2002 and 2008).
(PDF)

Figure S2 Seasonal (a) and long-term (b) trends of maximum temperature in Iquitos, Peru. In (a), annual trimesters (demarcated by dashed vertical lines) and the dengue season (red shaded area) are indicated. The blue line is the loess smoothed response with standard error, α = 0.5. In (b), solid line is the loess smoothed response with standard error (α = 0.5, blue envelope). The dashed line is for α = 0.1 with standard error (grey envelope, See Methods).
(PDF)

Figure S3 CCM and scatterplot at lag of highest |*r*| in Spearman and Pearson correlations for the whole year (large panels) and trimesters I and III.
(PDF)

Figure S4 Seasonal (a) and long-term (b) trends of mean temperature in Iquitos, Peru. In (a), annual trimesters (demarcated by dashed vertical lines) and the dengue season (red shaded area) are indicated. The blue line is the loess smoothed response with standard error, α = 0.5. In (b), solid line is the loess-smoothed response with standard error (α = 0.5, blue envelope). The dashed line is for α = 0.1 with standard error (grey envelope, See Methods).
(PDF)

Figure S5 CCM and scatterplot at lag of highest |*r*| in Spearman and Pearson correlations for the whole year (large panels) and trimesters I and III.
(PDF)

Figure S6 Seasonal (a) and long-term (b) trends of minimum temperature in Iquitos, Peru. In (a), annual trimesters (demarcated by dashed vertical lines) and the dengue season (red shaded area) are indicated. The blue line is the loess smoothed response with standard error, α = 0.5. In (b), solid line is the loess smoothed response with standard error (α = 0.5, blue envelope). The dashed line is for α = 0.1 with standard error (grey envelope, See Methods).
(PDF)

Figure S7 CCM and scatterplot at lag of highest |*r*| in Spearman and Pearson correlations for the whole year (large panels) and trimesters I and III.
(PDF)

Figure S8 Seasonal (a) and long-term (b) trends of DD_24_ in Iquitos, Peru. In (a), annual trimesters (demarcated by dashed vertical lines) and the dengue season (red shaded area) are indicated. The blue line is the loess smoothed response with standard error, α = 0.5. In (b), solid line is the loess smoothed response with standard error (α = 0.5, blue envelope). The dashed line is for α = 0.1 with standard error (grey envelope, See Methods).
(PDF)

Figure S9 CCM and scatterplot at lag of highest |*r*| in Spearman and Pearson correlations for the whole year (large panels) and trimesters I and III.
(PDF)

Figure S10 Seasonal (a) and long-term (b) trends of DTR in Iquitos, Peru. In (a), annual trimesters (demarcated by dashed vertical lines) and the dengue season (red shaded area) are indicated. The blue line is the loess smoothed response with standard error, α = 0.5. In (b), solid line is the loess smoothed response with standard error (α = 0.5, blue envelope). The dashed line is for α = 0.1 with standard error (grey envelope, See Methods).
(PDF)

Figure S11 CCM and scatterplot at lag of highest |*r*| in Spearman and Pearson correlations for the whole year (large panels) and trimesters I and III.
(PDF)

Figure S12 Seasonal (a) and long-term (b) trends of total weekly precipitation (cm) in Iquitos, Peru. In (a), annual trimesters (demarcated by dashed vertical lines) and the dengue season (red shaded area) are indicated. The blue line is the loess smoothed response with standard error, α = 0.5. In (b), solid line is the loess smoothed response with standard error (α = 0.5, blue envelope). The dashed line is for α = 0.1 with standard error (grey envelope, See Methods).
(PDF)

Figure S13 CCM and scatterplot at lag of highest |*r*| in Spearman and Pearson correlations for the whole year (large panels) and trimesters I and III.
(PDF)

Figure S14 Seasonal (a) and long-term (b) trends of weekly precipitation events in Iquitos, Peru. In (a), annual trimesters (demarcated by dashed vertical lines) and the dengue season (red shaded area) are indicated. The blue line is the loess smoothed response with standard error, α = 0.5. In (b), solid line is the loess smoothed response with standard error (α = 0.5, blue envelope). The dashed line is for α = 0.1 with standard error (grey envelope, See Methods).
(PDF)

Figure S15 CCM and scatterplot at lag of highest |*r*| in Spearman and Pearson correlations for the whole year (large panels) and trimesters I and III.
(PDF)

Figure S16 Seasonal (a) and long-term (b) trends of relative humidity in Iquitos, Peru. In (a), annual trimesters (demarcated by dashed vertical lines) and the dengue season (red shaded area) are indicated. The blue line is the loess smoothed response with standard error, α = 0.5. In (b), solid line is the loess smoothed response with standard error (α = 0.5, blue envelope). The dashed line is for α = 0.1 with standard error (grey envelope, See Methods).
(PDF)

Figure S17 CCM and scatterplot at lag of highest |*r*| in Spearman and Pearson correlations for the whole year (large panels) and trimesters I and III.
(PDF)

Figure S18 Seasonal (a) and long-term (b) trends of wind speed in Iquitos, Peru. In (a), annual trimesters (demarcated by dashed vertical lines) and the dengue season (red shaded area) are indicated. The blue line is the loess smoothed response with standard error, α = 0.5. In (b), solid line is the loess smoothed response with standard error (α = 0.5, blue envelope). The dashed line is for α = 0.1 with standard error (grey envelope, See Methods).
(PDF)

Figure S19 CCM and scatterplot at lag of highest |*r*| in Spearman and Pearson correlations for the whole year (large panels) and trimesters I and III.
(PDF)

Figure S20 Seasonal (a) and long-term (b) trends of Amazon River level (meters above sea level) in Iquitos, Peru. In (a), annual trimesters (demarcated by dashed vertical lines) and the dengue season (red shaded area) are indicated. The blue line is the loess smoothed response with standard error, α = 0.5. In (b), solid line is the loess smoothed response with standard error (α = 0.5, blue envelope). The dashed line is for α = 0.1 with standard error (grey envelope, See Methods).
(PDF)

Figure S21 CCM and scatterplot at lag of highest |*r*| in Spearman and Pearson correlations for the whole year (large panels) and trimesters I and III.
(PDF)

Figure S22 Seasonal (a) and long-term (b) trends of PC1 in Iquitos, Peru. In (a), annual trimesters (demarcated by dashed vertical lines) and the dengue season (red shaded area) are indicated. The blue line is the loess smoothed response with standard error, α = 0.5. In (b), solid line is the loess smoothed response with standard error (α = 0.5, blue envelope). The dashed line is for α = 0.1 with standard error (grey envelope, See Methods).
(PDF)

Figure S23 CCM and scatterplot at lag of highest |*r*| in Spearman and Pearson correlations for the whole year (large panels) and trimesters I and III.
(PDF)

Figure S24 Seasonal (a) and long-term (b) trends of PC2 in Iquitos, Peru. In (a), annual trimesters (demarcated by dashed vertical lines) and the dengue season (red shaded area) are indicated. The blue line is the loess smoothed response with standard error, α = 0.5. In (b), solid line is the loess smoothed response with standard error (α = 0.5, blue envelope). The dashed line is for α = 0.1 with standard error (grey envelope, See Methods).
(PDF)

Figure S25 CCM and scatterplot at lag of highest |*r*| in Spearman and Pearson correlations for the whole year (large panels) and trimesters I and III.
(PDF)

Figure S26 Seasonal (a) and long-term (b) trends of PC3 in Iquitos, Peru. In (a), annual trimesters (demarcated by dashed vertical lines) and the dengue season (red shaded area) are indicated. The blue line is the loess smoothed response with standard error, α = 0.5. In (b), solid line is the loess-smoothed response with standard error (α = 0.5, blue envelope). The dashed line is for α = 0.1 with standard error (grey envelope, See Methods).
(PDF)

Figure S27 CCM and scatterplot at lag of highest |*r*| in Spearman and Pearson correlations for the whole year (large panels) and trimesters I and III.
(PDF)

Figure S28 Summary of Spearman correlations.
(PDF)
