# Supplementary material for: Long-Term and Seasonal Dynamics of Dengue in Iquitos, Peru
Source: PLoS Negl Trop Dis. 2014 Jul 17;8(7):e3003. doi: 10.1371/journal.pntd.0003003 (PMC4102451; doi:10.1371/journal.pntd.0003003)
Supplement: Figure S1 — The local wavelet power spectrum (sqrt-transformed, dark red = high, dark blue = low) corresponds to the amount of variation in the signal that is explained by different periods (y-axis) as a function of time (x-axis). The global wavelet spectrum averages this variation across time. Throughout most of the time series there is a strong annual component with some sub-annual components driven by differences in epidemic timing and a double peak during the 2008–09 season. Multi-annual components are present, on the order of 2–3 years, but the length of the time series is too short to provide strong support. Accounting for edge effects, the periods that can be detected at a particular time lie above the thick black line (cone of influence). The areas of statistically significant power at the 5% level are contoured by the thin black lines. On the global wavelet spectrum, this threshold is indicated by the dashed line. (a) result assuming uncorrelated, white noise; (b) result assuming autocorrelated, red noise. Note in (b) the two novel serotype introductions are emphasized (2001–2002 and 2008). (PDF) [file pntd.0003003.s001.pdf]

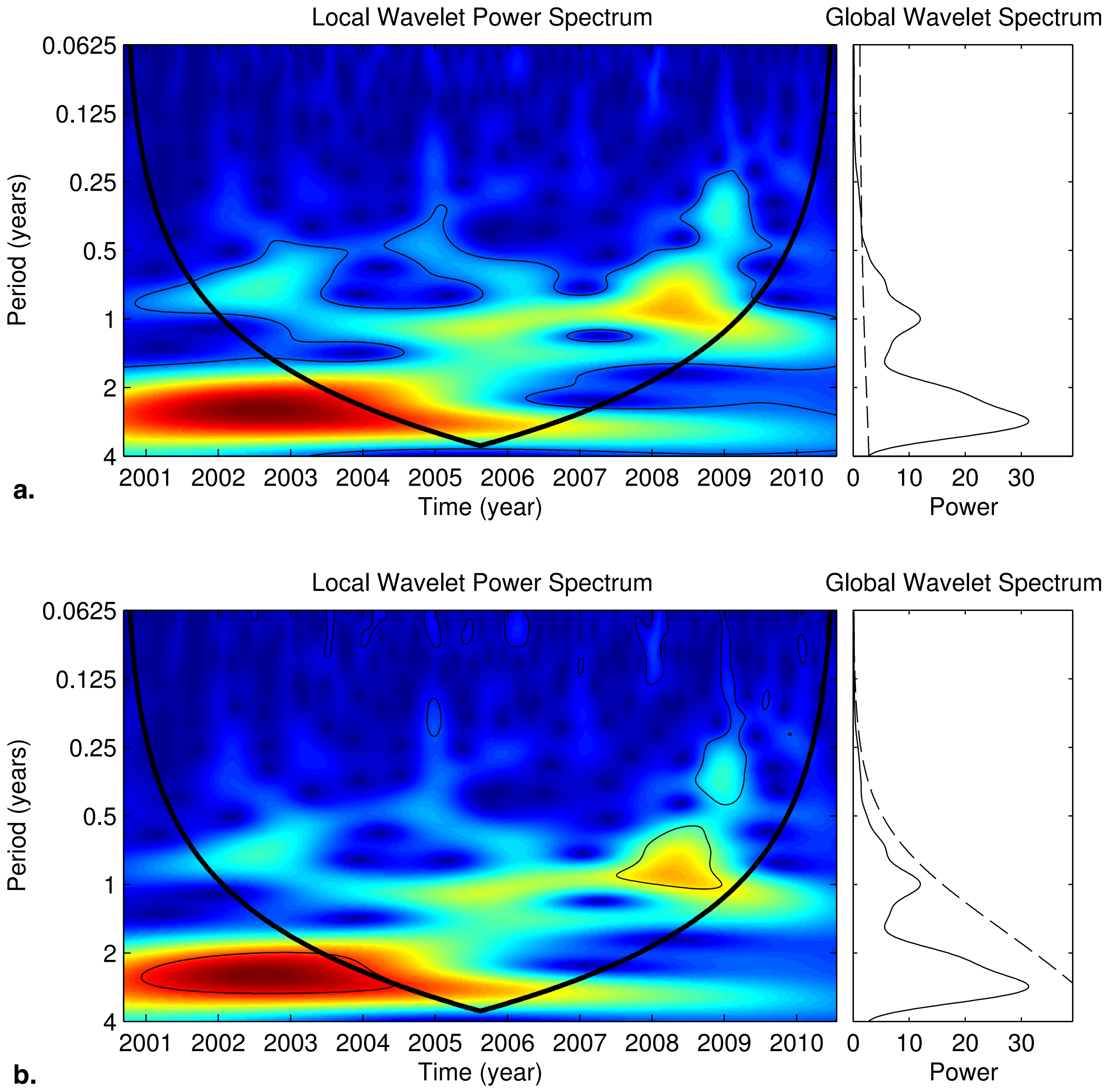

Figure S1: The local wavelet power spectrum (sqrt-transformed, dark red = high, dark blue = low) corresponds to the amount of variation in the signal that is explained by different periods (y-axis) as a function of time (x-axis). The global wavelet spectrum averages this variation across time. Throughout most of the time series there is a strong annual component with some sub-annual components driven by differences in epidemic timing and a double peak during the 2008-09 season. Multi-annual components are present, on the order of 2-3 years, but the length of the time series is too short to provide strong support. Accounting for edge effects, the periods that can be detected at a particular time lie above the thick black line (cone of influence). The areas of statistically significant power at the 5% level are contoured by the thin black lines. On the global wavelet spectrum, this threshold is indicated by the dashed line. (a) result assuming uncorrelated, white noise; (b) result assuming autocorrelated, red noise. Note in (b) the two novel serotype introductions are emphasized (2001-2002 and 2008).
