# Supplementary material for: Long-Term and Seasonal Dynamics of Dengue in Iquitos, Peru
Source: PLoS Negl Trop Dis. 2014 Jul 17;8(7):e3003. doi: 10.1371/journal.pntd.0003003 (PMC4102451; doi:10.1371/journal.pntd.0003003)
Supplement: Figure S6 — Seasonal (a) and long-term (b) trends of minimum temperature in Iquitos, Peru. In (a), annual trimesters (demarcated by dashed vertical lines) and the dengue season (red shaded area) are indicated. The blue line is the loess smoothed response with standard error, α = 0.5. In (b), solid line is the loess smoothed response with standard error (α = 0.5, blue envelope). The dashed line is for α = 0.1 with standard error (grey envelope, See Methods). (PDF) [file pntd.0003003.s006.pdf]

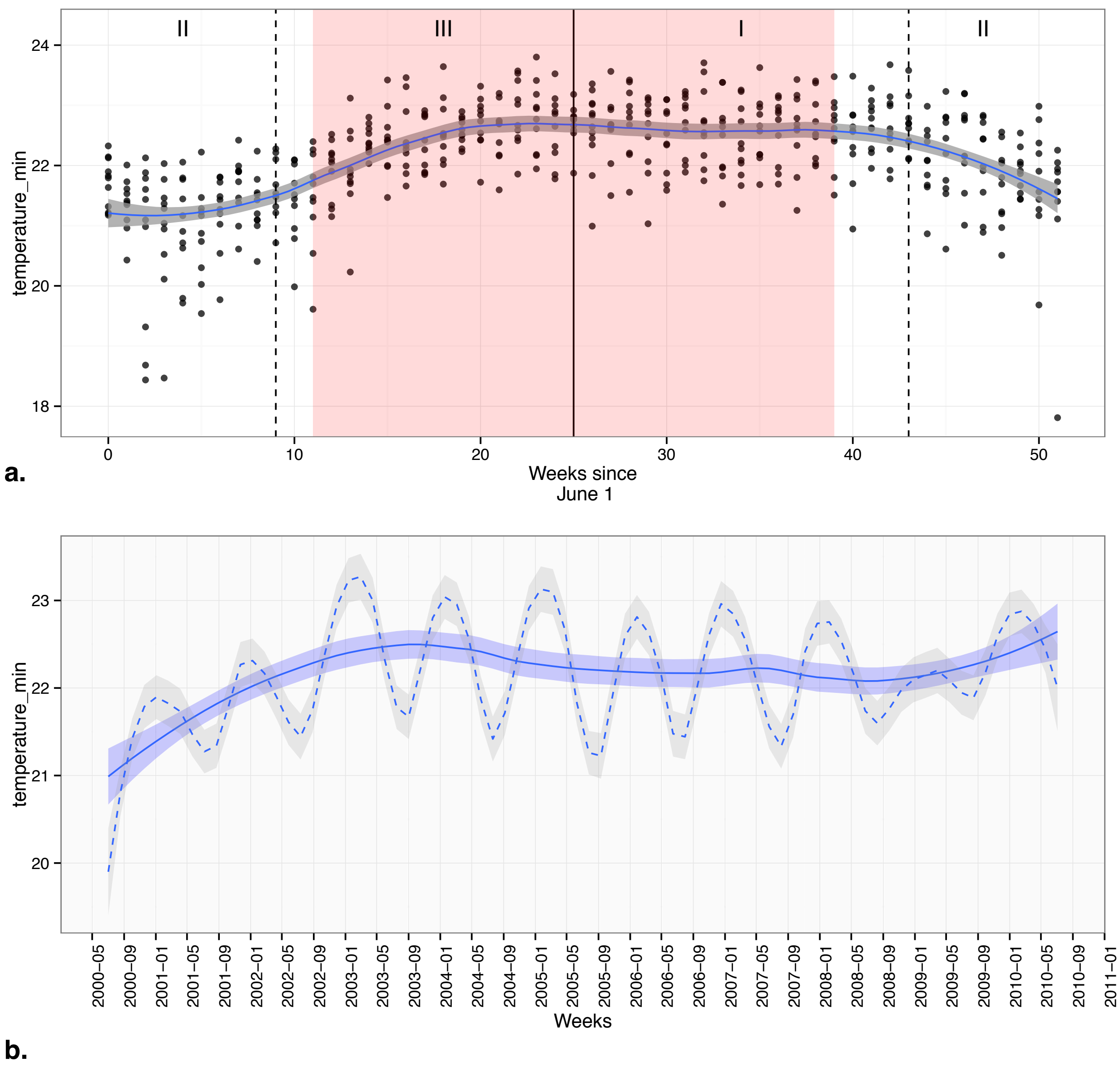

Figure S6: Seasonal (a) and long-term (b) trends of minimum temperature in Iquitos, Peru. In (a), annual trimesters (demarcated by dashed vertical lines) and the dengue season (red shaded area) are indicated. The blue line is the loess smoothed response with standard error, $\alpha = 0.5$. In (b), solid line is the loess smoothed response with standard error ($\alpha = 0.5$, blue envelope). The dashed line is for $\alpha = 0.1$ with standard error (grey envelope, See Methods).
