# Supplementary material for: Long-Term and Seasonal Dynamics of Dengue in Iquitos, Peru
Source: PLoS Negl Trop Dis. 2014 Jul 17;8(7):e3003. doi: 10.1371/journal.pntd.0003003 (PMC4102451; doi:10.1371/journal.pntd.0003003)
Supplement: Figure S9 — CCM and scatterplot at lag of highest |r| in Spearman and Pearson correlations for the whole year (large panels) and trimesters I and III. (PDF) [file pntd.0003003.s009.pdf]

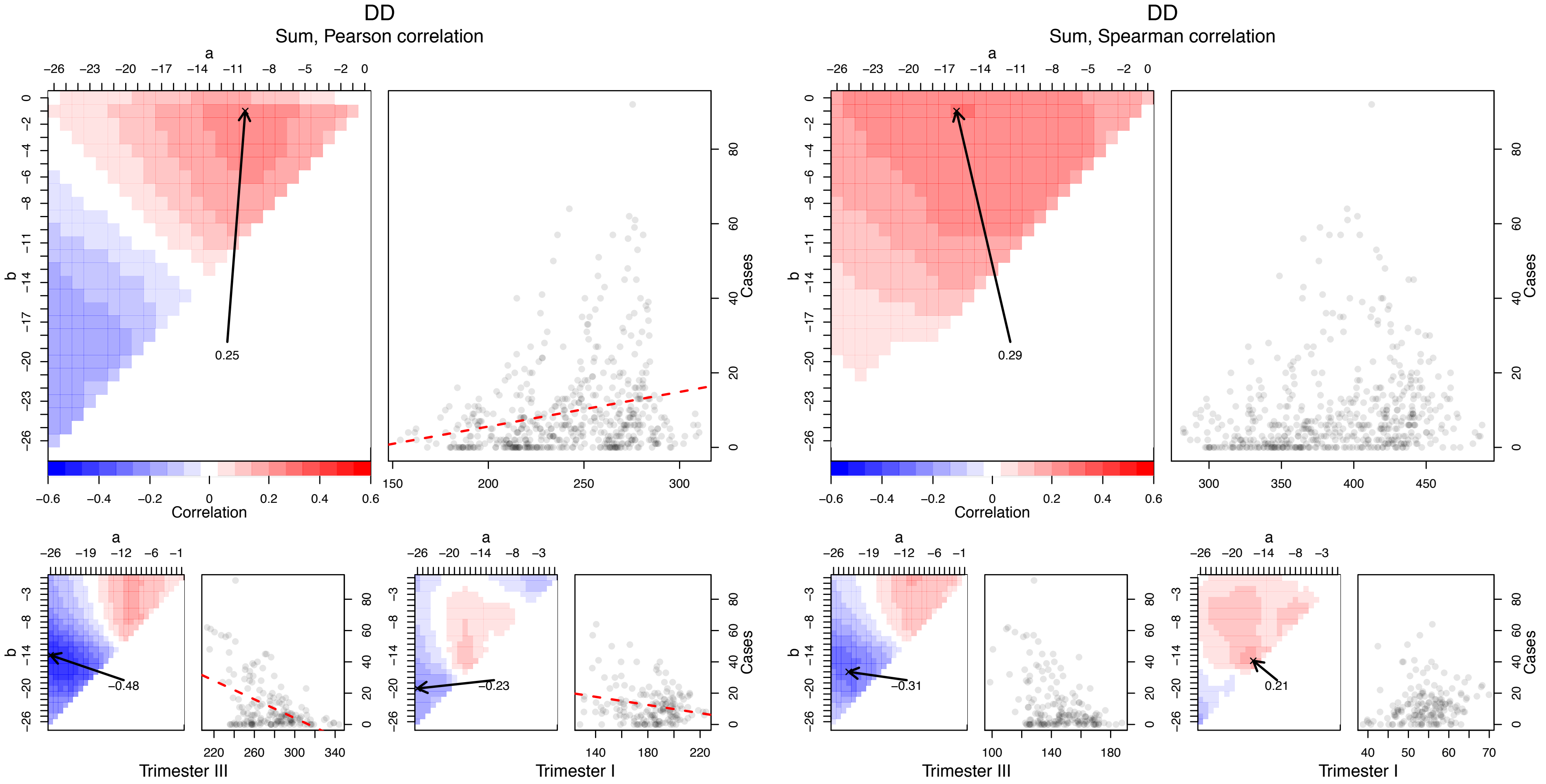

Figure S9: CCM and scatterplot at lag of highest |r| in Spearman and Pearson correlations for the whole year (large panels) and trimesters I and III.
