# Supplementary figures and images for: Long-Term and Seasonal Dynamics of Dengue in Iquitos, Peru
Source: PLoS Negl Trop Dis. 2014 Jul 17;8(7):e3003. doi: 10.1371/journal.pntd.0003003 (PMC4102451; doi:10.1371/journal.pntd.0003003)

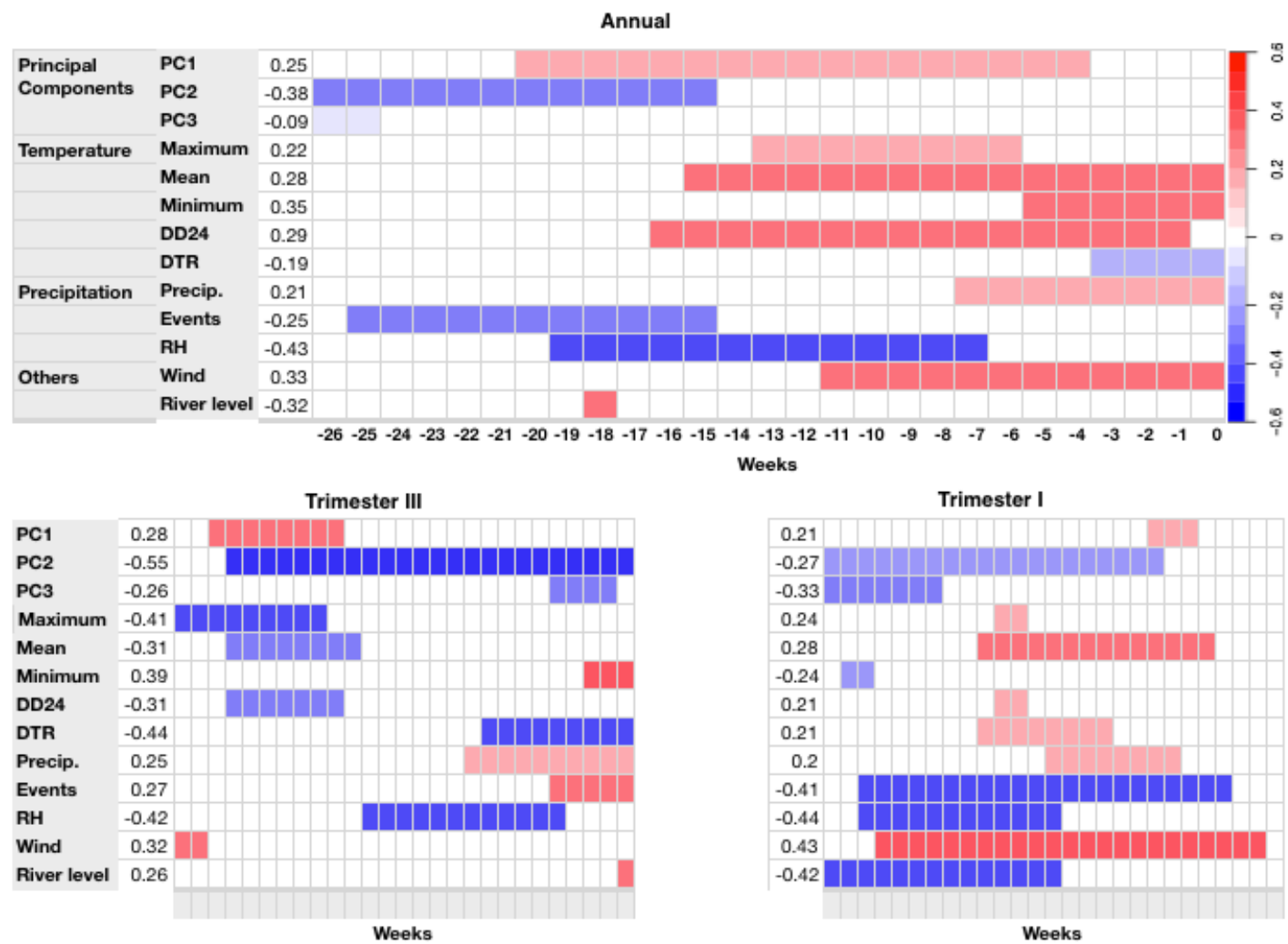

**Figure S28:** Summary of Spearman correlations.

Supplement: Figure S28 — Summary of Spearman correlations. (PDF) [file pntd.0003003.s028.pdf]
